# Hypsometric changes in urban areas resulting from multiple years of mining activity

**DOI:** 10.1038/s41598-022-06847-8

**Published:** 2022-02-22

**Authors:** Maksymilian Solarski, Robert Machowski, Mariusz Rzetala, Martyna A. Rzetala

**Affiliations:** 1 Institute of Social and Economic Geography and Spatial Management, Faculty of Natural Sciences, University of Silesia in Katowice, Bedzinska 60, 41-200 Sosnowiec, Poland; 2 Institute of Earth Sciences, Faculty of Natural Sciences, University of Silesia, Bedzinska 60, 41-200 Sosnowiec, Poland

**Keywords:** Environmental sciences, Natural hazards

## Abstract

The impact of multiple years of underground mining of minerals on changes in the elevation of an urban area has been evaluated using the case study of Bytom in southern Poland. Between 1883 and 2011, that city experienced changes in absolute minimum (from 250.0 to 243.0 m a.s.l.) and maximum (from 340.0 to 348.4 m a.s.l.) elevations. During that period, the difference between minimum and maximum elevations increased from 90.0 to 105.4 m. The consequence of underground mining has been the formation of extensive subsidence basins with a maximum depth of 35 m. Where the terrain became raised, its elevation rose most commonly by 1.1 m to 5.0 m, with maximum increase in elevation caused by human activity amounting to 35 m. The rate of anthropogenic subsidence in the city between 1883 and 2011 averaged 43 mm/year (5.5 m over the study period).

## Introduction

Humans have mined mineral resources for many thousands of years. Evidence of various minerals being extracted, both in opencast and underground mines, has been found all over the world. In the past, this type of activity was limited to small areas and usually did not cause significant environmental changes^1–4^. It was not until the industrial revolution of the eighteenth and nineteenth centuries, which brought technological advances, that mining activity developed on an industrial scale^5^.

Underground extraction and processing of minerals usually results directly and indirectly in the degradation of the geographical environment^6–8^. Its most common negative impact involves changes in terrain elevation. An unpredictable consequence of this activity are subsidences which form above the mineral deposits mined and in their close vicinity^9–15^. In scientific discussions concerning mining damage, land subsidence is the most frequent subject^16–21^. On the other hand, there are almost no comprehensive studies which would simultaneously provide information on changes in land relief which manifest themselves by an increase in the absolute elevation of the area in question. A general overview of this subject was presented for the entire Ruhr area in Germany where the role of humans in shaping the relief of that area was analysed^22,23^. An attempt to assess the geomorphological effects of mining activities was also made in relation to small study areas in southern Poland^24^.

Subsidence is a slow process which virtually always results in changes in the environment and has both social and economic consequences^25^. On the other hand, storage of waste rock (mineral waste generated in the process of extracting raw materials) in the form of heaps and dumps is usually planned in advance and fully controlled by man^26–29^. As it is impossible to completely eliminate subsidence, mines are located outside urban areas^30,31^. However, if the mining fields already extend to urban areas, mining is not carried out under built-up areas, which are protected by so-called safety pillars.

**Figure 1:**
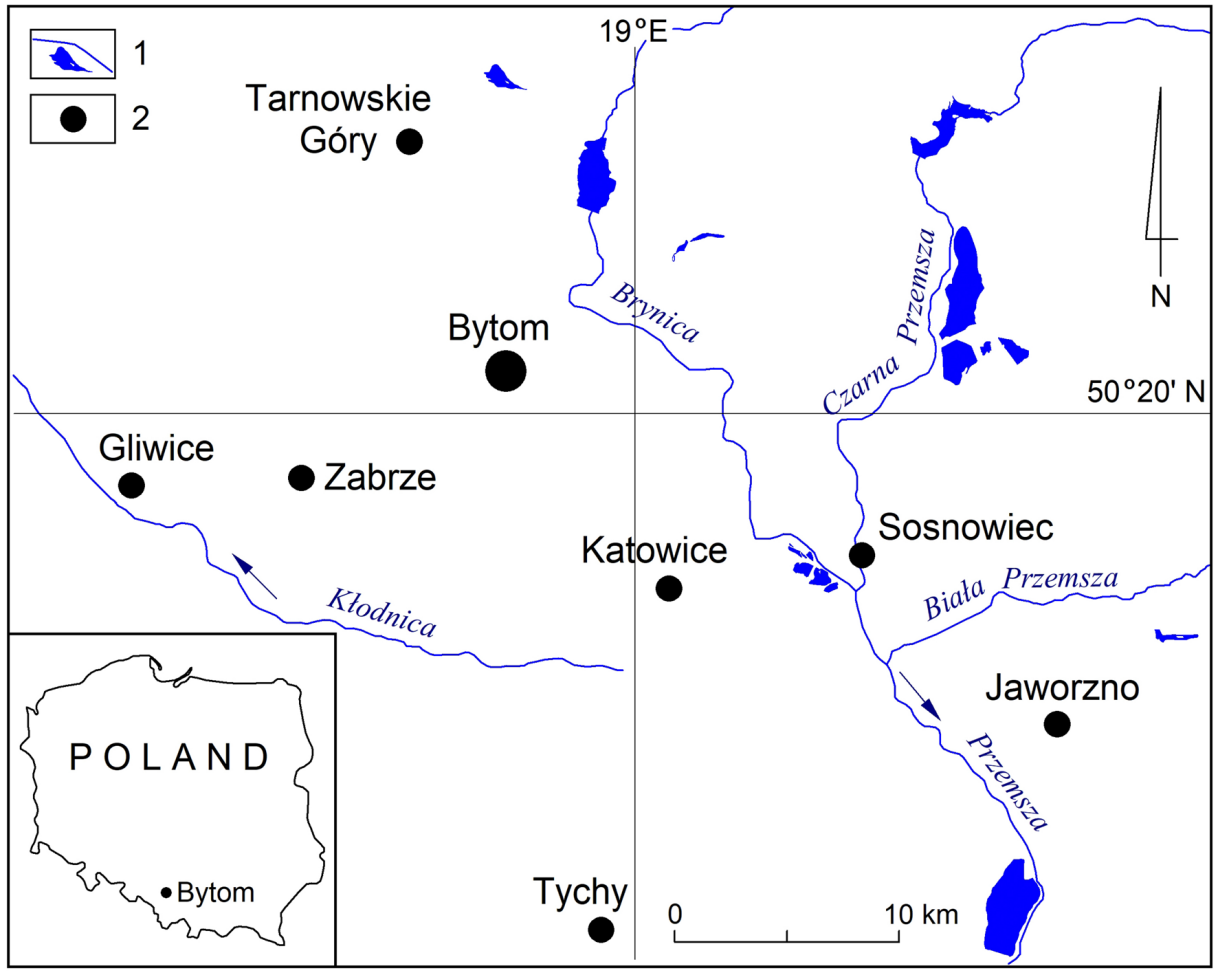
Study area location: 1—water courses and water reservoirs; 2—major cities.

The Upper Silesian Coal Basin in southern Poland, which is among the largest mining districts in Europe, is unique in this respect. Of the dozen or so cities present in the area, Bytom has been particularly affected by the extreme changes in terrain elevation caused by deep mining (Fig. 1). As a consequence of underground mining activities carried out within city boundaries for several centuries, many different types of artificial land forms have appeared in its landscape. Among them, both convex (slag and other types of heaps, embankments, mounds, etc.) and concave (mineral workings, subsidence basins and hollows, etc.) forms can be found, which have significantly contributed to obliterating the natural features of the terrain.

The aim of this study was to assess the influence of many years of underground extraction of minerals on changes in elevation in an urban area. Owing to the fact that the intensity of mining activity, and thus also its effects visible on the surface, varied over time, it was necessary to use different time periods for which detailed studies were performed.

## Materials and methods

### Study area

Bytom is located within the Upper Silesian Coal Basin. Within its current administrative borders, the area of the city is 69.74 km^2^. Geological formations rich in silver, lead and zinc ores, but primarily in hard coal, are present in the study area. On the land surface, Quaternary sediments dominate. In the southern part of the city, these include sands, loams and weathered silts formed on glacial tills. The middle sector is mainly covered by sands and gravels of fluvioglacial origin. In the northern part of Bytom, glacial tills have generally formed, and there are also deposits of terminal moraine sands, gravels, boulders and glacial tills in places. Outcrops of older geological formations are present directly on the surface throughout the city, mostly in the form of small lenses. These types of rock are represented by Triassic dolomites and limestones, limestones and marls, ore-bearing dolomites as well as limestones. In the southern part of the city, directly under the layer of Quaternary sediments with a thickness ranging from 40 to 80 m, Carboniferous rock formations appear, which contain coal deposits. In general, Carboniferous sediments in the Bytom area are covered by Middle and Lower Triassic rocks. Owing to the presence of numerous tectonic faults, the position of the topmost Carboniferous layer is variable and ranges from approximately 80 m a.s.l. to approximately 210 m a.s.l. The hard coal deposits mined contain rocks originating from the Upper Carboniferous. The Mudstone Series includes mudstones, claystones, sandstones and hard coal (Lower Westphalian). The Upper Silesian Sandstone Series consists of sandstones, conglomerates, claystones, mudstones and shales as well as hard coal (Upper Namur). The Paralic Series includes claystones, mudstones, sandstones and also hard coal (Lower Namur)^32^.

### Research methods

Historical maps may serve as a valuable source of information on the changes in the natural environment that have occurred in the last few hundred years. Terrain relief started to be presented on topographic maps using contour intervals at the end of the nineteenth century. To study the transformations of this element of the natural environment, 1:25,000 Prussian *Messtischblätter* maps from the end of the nineteenth century^33^ and 1:10,000 Polish topographic maps from the end of the twentieth century were used^34^. The archival Prussian maps were drawn up using the plane-table survey method by qualified military cartographers. They use a polyhedral projection based on the Bessel ellipsoid. The main contour interval on these maps is 20 m, with auxiliary isolines added every 1.25 m, 2.50 m and 3.75 m in the plains. According to W. Jankowski^35^, the average error of point location on these maps, calculated on the basis of geographical coordinates, is (plus or minus) 20.1 m compared to contemporary maps. On the basis of his studies of selected *Messtischblätter* map sheets^33^, Konias^36^ found that the average distance error on these maps is (plus or minus) 41.0 m compared to contemporary maps, which corresponds to 1.2 mm given the map’s scale. Thus, these maps are fairly good in cartometric terms in relation to those produced at the end of the twentieth century. The contemporary Polish maps were produced using the 92 coordinate system and the Gauss-Krüger projection. The main contour interval on these maps is 10 m, with a secondary interval every 1.25 m.

The analysis of hypsometric changes was preceded by the calibration of archival topographic materials to fit the modern coordinate system (imposing a modern coordinate system on historical maps). Calibration enables the map in question to be located spatially and minimises the distortions resulting from material deformations, thus enabling various spatial analyses to be performed on the basis of the map and comparisons to be made between the state of affairs presented on the map and the contemporary one. GIS tools make it possible to rectify maps using contemporary topographic elements; in this case, by analogy, topographic points on an archival map (e.g. road junctions and churches) are given the coordinates read from contemporary maps. The archival maps were fitted using first-degree affine transformation using several dozen chaotically distributed characteristic points (per map sheet). Calibration was performed using the ArcMap 10 software.

In order to determine the changes in land relief in Bytom, two digital terrain models were produced on the basis of archival maps (1883—contour lines from the *Messtischblätter* maps from the end of the nineteenth century) and contemporary maps^33,34^. The terrain models were produced in the Map Info Professional 10.0 software using the Vertical Mapper device. The models were based on the linear vectorisation of contour lines, which were subsequently converted into points situated at certain heights above sea level. The points created in this manner were interpolated using the natural neighbour method. On the basis of the resulting terrain models, terrain profiles, cartograms of relief intensity and of changes in relief intensity were produced, and changes in height relationships, differences in height and slopes within the city area were determined in quantitative terms. The final stage of the analysis was to produce a map of changes in land relief. For this purpose, a simple raster map algebra was applied using the Vertical Mapper overlay: A (1883) – B (1994) = C (the magnitude of subsidence or “growth” in land relief in the period between 1883 and 1994).

Archival maps are a reliable source of knowledge about landform changes in historical times^37–40^). The Prussian Meßtischblätter maps used in this study are characterised by very high detail and accurate topography and are among the best sources of information on the spatial transformations (e.g. land use, changes in water relations and in land relief) that have taken place within the last 120 years^22,23,41,42^. According to Harnischmacher and Zepp^23^ who studied relief changes in the Ruhr, almost 99% of data from this area had an elevation difference error of less than 2 m, and only slightly more than 1% had an error greater than 2 m. The study by Dulias^43^, which covers the entire area of the Upper Silesian Coal Basin, demonstrates that the maximum error was ± 0.45 m. The comparison between the two terrain models developed for that study shows that the maximum differences in terrain elevation in the area where no changes in relief occurred due to human activity during the studied period (wooded areas in the northern part of the city) do not exceed ± 1.5 m.

The most recent terrain model was compiled using data from aerial laser scanning (LIDAR), which was carried out on 18 April 2011. Elevation data acquired by way of laser scanning exhibit very high spatial resolution, as there are eight measurement points per square metre and the average absolute elevation measurement error does not exceed 0.15 m^44^.

## Results

**Figure 2:**
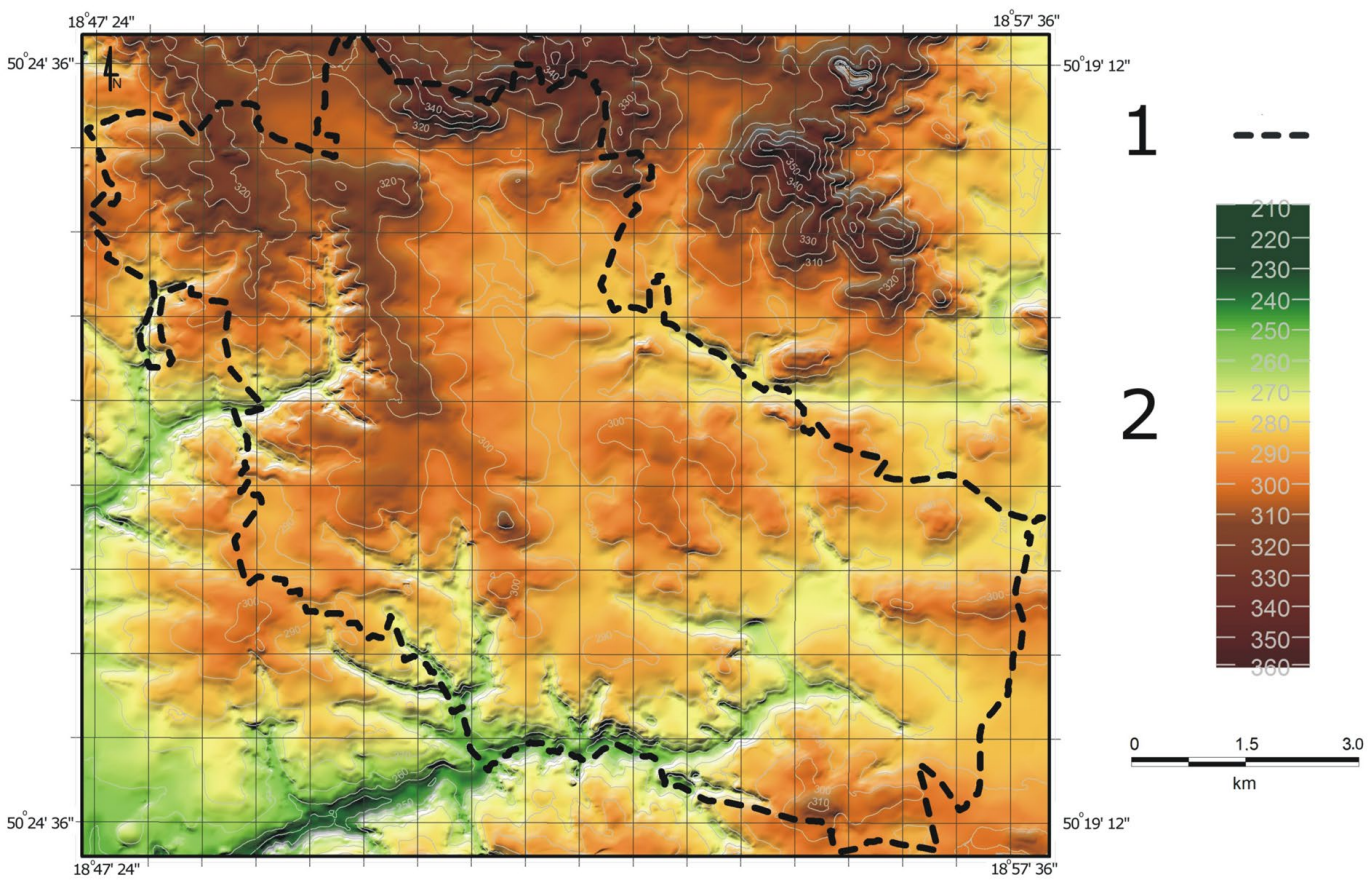
Land relief within the city of Bytom at the end of the nineteenth century (within present-day city limits): 1—city boundary; 2—absolute elevation [m a.s.l.].

**Figure 3:**
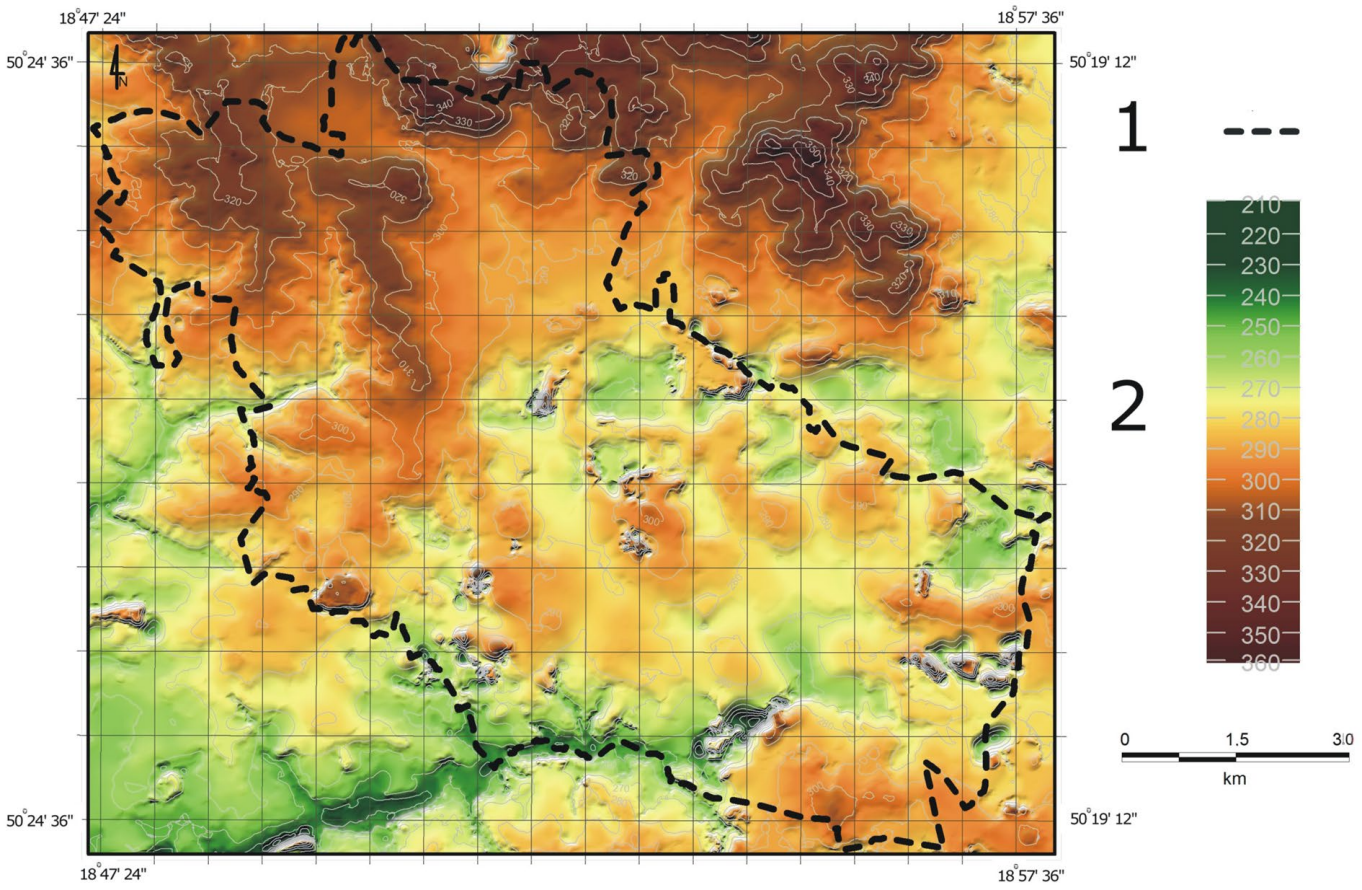
Land relief within the city of Bytom at the end of the twentieth century (within present-day city limits): 1—city boundary; 2—absolute elevation [m a.s.l.].

At the beginning of the study period, the entire city was essentially on a slope that descended from the north towards the south (Fig. 2). In the north of Bytom, there were areas with a maximum elevation of 340 m a.s.l., while in the southern part of the city (which included the river valley) the absolute elevation reached 250 m a.s.l. At the end of the nineteenth century, differences in elevation within town limits amounted to 90 m. The average elevation of the city was 292.6 m a.s.l.; land ranging in elevation from 280.1 m to 310.0 m a.s.l. accounted for almost 80% of the area studied (Table 1). At the end of the twentieth century, there was a noticeable increase in the proportion of the lowest areas, i.e. those situated below 280.1 m a.s.l. (Fig. 3). At that time, areas located at elevations above 340 m a.s.l., which until then had not been present in the city area as a result of natural geomorphological conditions, were identified as well. Those changes were at the expense of the areas ranging from 280.1 m to 310.0 m a.s.l. in elevation whose share in the total area analysed decreased by as much as 19%. In 1994, the elevation difference between the two points with extreme elevations increased to 102.8 m. At that time, the average absolute elevation was 287.8 m. At the beginning of the second decade of the twenty-first century, there were slight changes in the distribution of individual elevation ranges. Again, the proportion of land situated at the lowest elevation increased (by 5.3% compared to 1994), while a slight decrease in the other ranges was recorded. By 2011, the average absolute elevation had decreased by a further 0.7 m to 287.1 m a.s.l. and the difference between maximum and minimum elevation had increased to 105.4 m.

**Table 1:** Differences in elevation within the Bytom city area in the years 1883–2011.

Elevation [m a.s.l.]	Share in total area of the city					
	1883		1994		2011	
	[ha]	[%]	[ha]	[%]	[ha]	[%]
240.0–250.0	7.0	0.1	34.9	0.5	10.5	0.1
250.1–260.0	69.7	1.0	174.4	2.5	32.8	0.5
260.1–270.0	174.4	2.5	502.1	7.2	606.7	8.7
270.1–280.0	606.7	8.7	1464.5	21.0	1896.5	27.2
280.1–290.0	2350.2	33.7	2001.5	28.7	2020.4	29.0
290.1–300.0	1855.1	26.6	1332.0	19.1	1129.8	16.2
300.1–310.0	1276.2	18.3	822.9	11.8	730.9	10.5
310.1–320.0	495.2	7.1	474.2	6.8	438.0	6.3
320.1–330.0	90.7	1.3	132.5	1.9	87.9	1.2
330.1–340.0	48.8	0.7	27.9	0.4	20.9	0.3
340.1–350.0	0.0	0.0	7.0	0.1	0.0	0.0
Ʃ	6974.0	100.0	6974.0	100.0	6974.0	100.0

At the end of the nineteenth century, Bytom was mostly flat: land with a gradient of up to 3° accounted for slightly more than 85% of city area. Areas with gradients ranging from 3.1° to 9.0° occupied approximately 14%. Areas with gradients above 20° were virtually absent, and those with gradients between 9.1° and 20° represented less than a percent of the total area of the city (Table 2). The average gradient of the area analysed at that time was 1.7°. At the end of the twentieth century, areas with gradients of up to 3° still prevailed in Bytom, occupying 5454 km^2^, i.e. 78.2% of the total area analysed. Within that range, the proportion of flat land (< 1.0°) decreased significantly (by 13.7%). In 1994, an increase in the share of land with steeper gradients in each of the ranges was noted, and areas with gradients greater than 20° could be identified as well. In the early 2010s, there was a significant decrease in the share of areas with gradients of up to 3º, which accounted for a total of 43.1%. The share of flat areas (< 1.0°) decreased by nearly 20% in just 17 years. During that time, the city saw a significant increase in the share of areas with gradients greater than 3°. There was also a marked increase in areas with the greatest gradients—to 9.5% in the (9.1°–20.0°) range and to 6.3% above 20°. The average gradient in Bytom at that time was 5.8°.

**Table 2:** Land gradients in the Bytom area from 1883 to 2011.

Gradient [°]	Share in total area of the city [%]			1883–2011
	1883	1994	2011	Δ%
< 1.0º	42.0	28.3	8.4	33.6
1.0º–3.0º	43.1	49.9	34.7	8.4
3.1º–5.0º	10.0	14.5	24.3	− 14.3
5.1º–9.0º	4.1	5.2	16.8	− 12.7
9.1º–20.0º	0.8	1.5	9.5	− 8.7
> 20.0º	0.0	0.6	6.3	− 6.3

## Discussion

The study area is located within one of the oldest and largest mining districts in Europe—the Upper Silesian Coal Basin in southern Poland. The intensive economic development of this area was connected with the discovery and extraction of mineral resources such as iron, silver, lead and zinc ores, but especially rich deposits of hard coal. In the eastern part of the Basin, one of the thickest hard coal seams in the world (Reden 510) is present with a thickness of up to 24 m^45^. Those rich deposits allowed the mining industry to flourish, followed by the processing industry. A number of changes have taken place in the geographical environment of the area as a result of many centuries of economic activity. The most spectacular effects are visible in land forms and as differences in elevation, in the form of continuous deformations: subsidence basins, which are an unintended consequence of underground mining of the aforementioned mineral resources using the so-called caving method^43,46^. This phenomenon is not limited to the mining area itself, but also affects areas in its immediate vicinity^47^. It is estimated that the subsidence caused by deep mining will eventually affect around 1000–1500 km^2^ within the Upper Silesian Coal Basin^48^. However, already in the early 1990s, the extent of this type of deformation reached 1185 km^2^^49,50^. Although depressions of 0.5–1.0 m prevailed, in some places the maximum depth observed in the centres of individual subsidence basins reached several dozen metres^43,47^. This concerned several cities and towns situated within the Basin. In the northern part of Ruda Śląska, a maximum subsidence of 24–28 m was found. Subsidence basins exceeding 20 m which had formed as a result of mining activities were also found in the southern suburbs of Rybnik, within the area from which hard coal was extracted by the “Jankowice” and “Chwałowice” mines^43^. Man-made depressions appeared in large numbers in several other cities and towns within the Basin. The deepest subsidence basin identified in Zabrze reached the depth of 12 m at the turn of the twenty-first century^51^. The magnitude of subsidence has been much smaller in Sosnowiec, where in the early 2000s it remained at a level of a few metres^52^.

**Figure 4:**
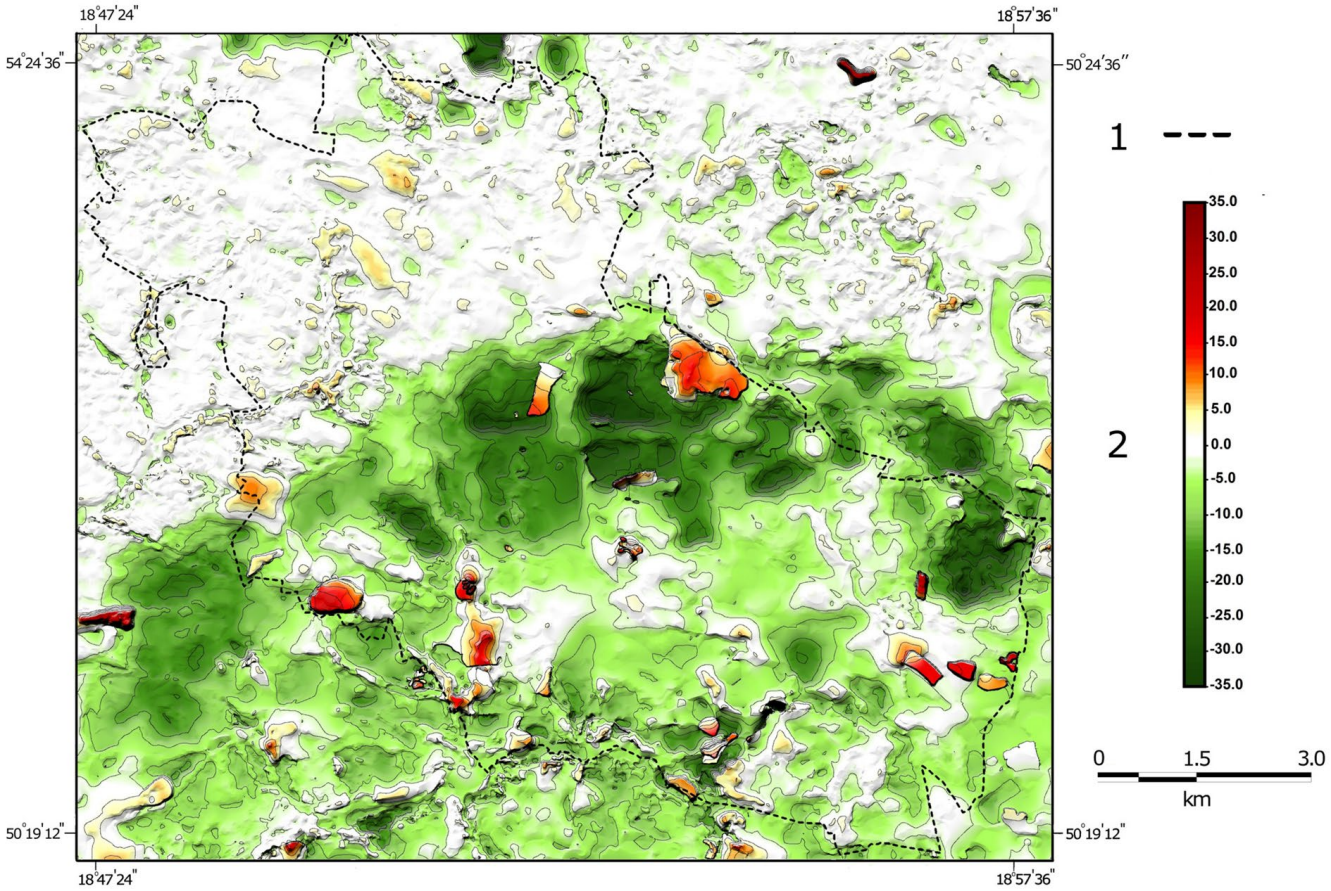
Changes in relative elevation within Bytom between 1883 and 1994: 1—city boundary; 2—elevation changes at selected height control points; 3—elevation changes within city limits.

Within Bytom, there are numerous and extensive depressions which reflect the deep mining of mineral resources. Such negative landforms have for many years been accompanied by waste dumps generated during the extraction and enrichment of such minerals. By 2011, as a result of mining subsidence, the elevation of the entire city of Bytom decreased by 5.5 m on average. As a result, it can be concluded that the anthropogenic subsidence rate in the period in question averaged 43 mm per year. It should be noted that mining subsidence has not affected the northern part of the city at all, which indicates that the magnitude of subsidence has been much greater in the areas which were affected by mining. Changes in relative elevation have generally affected the central and southern districts of the city (Fig. 4). In total, man-made land subsidence affected 64.5% of Bytom’s area. Most often, man-made depressions in the city reached a depth of 1.1 m to 5.0 m; those were found in just over 28% of the city area (Table 3). Depressions with a depth of up to 10 m make up a smaller share of over 17%. Land subsidence exceeding 10 m covered in total 19.2% of Bytom’s area. The greatest land subsidence could be found in three parts of the city^53^. The deepest subsidence basin emerged in the part of the study area located to the east of the centre, where depressions reached 35 m by the end of the twentieth century. In the central part of the study area, they were up to 30 m. On the other hand, in the south-eastern part of the city, a subsidence basin up to 34 m deep appeared. Numerous subsidence basins with maximum depths often exceeding 20 m appeared within the city limits in places where mineral resources had been extracted for many years. Such large-scale subsidence spreading into inhabited areas is virtually unheard of in other cities and towns in the region.

**Table 3:** Land elevation changes in the Bytom area between 1883 and 1994.

No.	Changes in elevation	Share [%] in the city area
1	> 20.0 m	0.1
2	10.1 m–20.0 m	1.4
3	5.1 m–10.0 m	2.1
4	1.1 m–5.0 m	10.6
5	− 1.0 m to + 1.0 m	21.3
6	− 1.1 m to − 5.0 m	28.2
7	− 5.1 m to − 10.0 m	17.1
8	− 10.1 m to − 20.0 m	14.6
9	− 20.1 m to − 30.0 m	4.3
10	< -30.0 m	0.3

Scientific reports from different parts of the world concerning mining subsidence have been quite numerous, but in no case have such large changes in elevation in urban areas been identified. Phenomena of this type are most often found in agricultural or undeveloped areas. Much of information of this type comes from China where coal mining is carried out in numerous large-scale mines. As reported by Quanyuan et al.^29^, mining subsidence in the built-up areas of Longkou city after 20 years of coal mining was only identified in an area of 5.7 hectares. The maximum depth of subsidence basins inventoried throughout the Longkou region only exceeds 5 m.

Among the many factors that influenced the changes in elevation in the city in the period from 1880 to 2010, undoubtedly the most significant were the continuous and discontinuous processes (the subsidence and caving in induced by mining activities) as well as the formation of waste rock dumps. Changes in elevation resulting from the emergence of various concave (e.g. pits, funnels, levelled surfaces) and convex forms (e.g. embankments, piles, dykes, mounds) are also related to mining activities. Similar forms are caused by the activity of other industries or services, or even agriculture, although their impact on elevation changes is much smaller in terms of the vertical range. The impact of these factors on the elevation is often compensated (e.g. when subsidence basins are backfilled with waste rock), but in some places they become particularly evident due to the absence of activities that would offset the progressing changes. Negative changes in elevation caused by other factors, e.g. progressing groundwater extraction (mining drainage, water supply), cannot be ruled out as well, although this problem has not been reported in the literature to date. As a result of the peculiar geological structure of the area, with the substrate made of solid rocks (e.g. dolomites, shales, quartzites) and the scant presence of loose surface formations, the problem of land subsidence induced by water extraction does not occur in practice. It is also impossible to compare the administrative unit examined with other agglomerations around the world which struggle with land subsidence caused by intensive groundwater extraction from loose formations. The land subsidence caused by this factor affects, for instance, Mexico City^54,55^, some urban areas in the United States^54^, some cities in China^55–57^ and many other cities worldwide, e.g. Jakarta or Tokyo^58,59^.

The mining subsidence that occurred in the Bytom area resulted in considerable damage to residential buildings. This occurred in the Karb district in particular: 28 residential buildings were damaged by subsidence and demolished as a result^60^.

**Figure 5:**
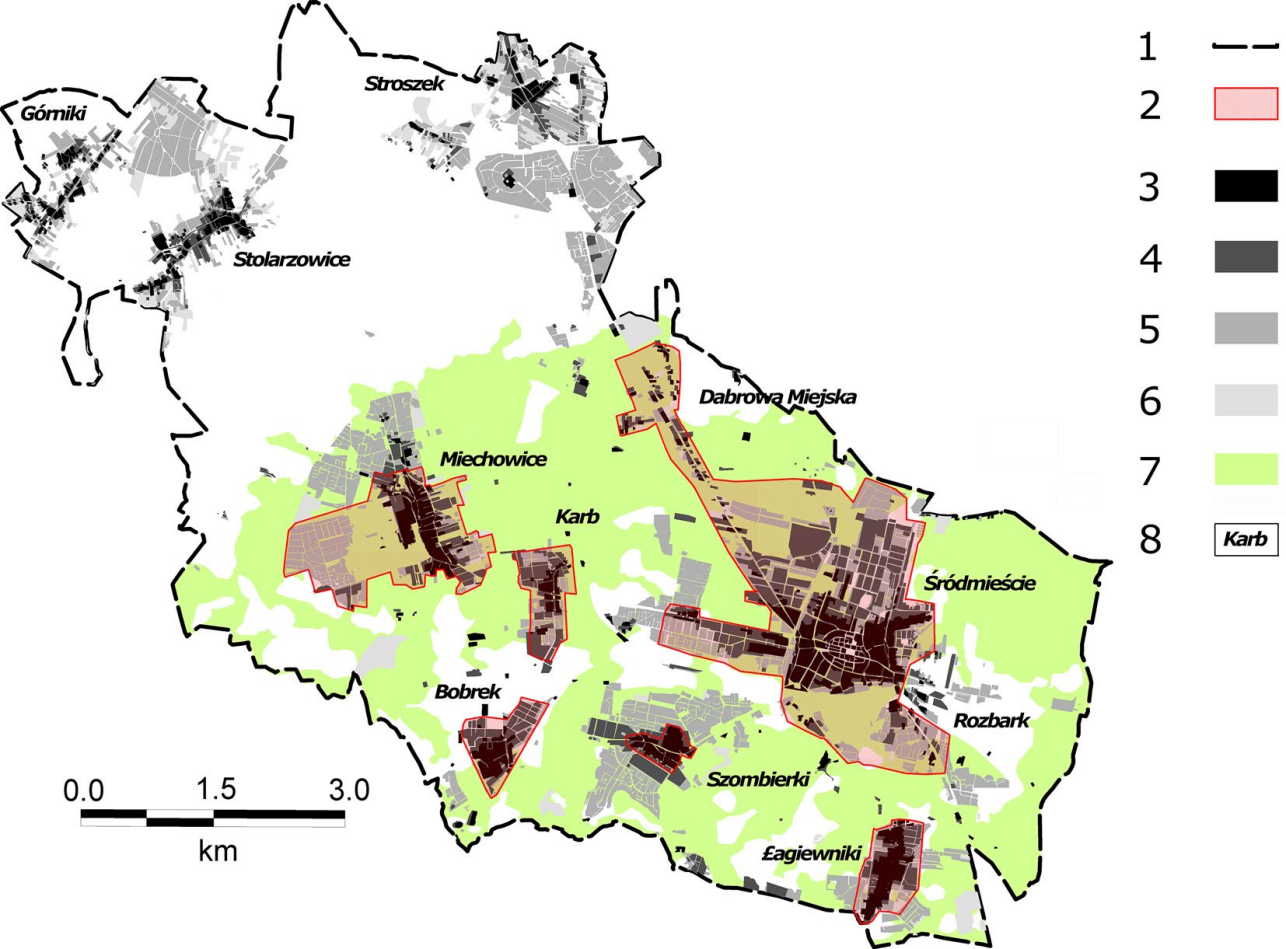
Land subsidence in the Bytom area in the years 1883–2011: 1—contemporary city limits; 2—built-up areas where mining damage and demolitions occurred; 3—built-up areas (as at 1883); 4—built-up areas (as at 1934); 5—built-up areas (as at 1994); 6—built-up areas (as at 2011); 7—range of land subsidence in the period 1883–2011; 8—city districts.

**Figure 6:**
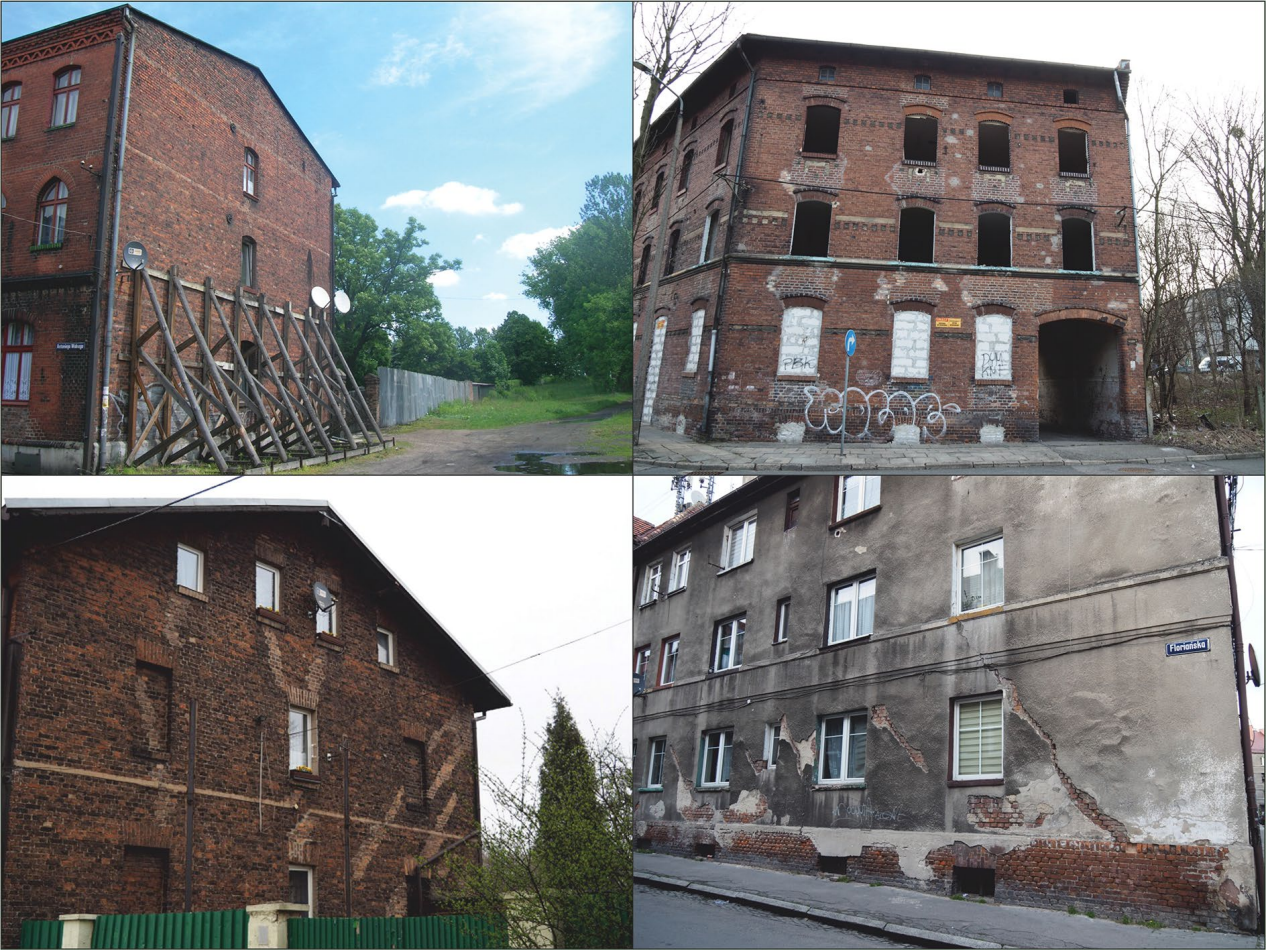
Examples of damage to Bytom’s urban fabric as a consequence of land subsidence (photo: M. Solarski).

Land subsidence in Bytom is particularly pronounced in urbanised areas whose share in the total area examined has been steadily increasing (1883–270.5 ha, 1934–567.7 ha, 1994–1217.0 ha, 2011–1387.0 ha). Land surface deformation and the related structural damage result in concrete economic losses (Figs. 5, 6), e.g. damage and destruction of buildings, structural and operational damage to various types of transport routes, flooding of land including city squares and human settlements, water reservoirs falling into disuse, and the need to vertically extend and frequently renovate watercourse embankments and hydraulic structures. This damage to the urban fabric is demonstrated by the fact that mining damage affects 940.6 ha of land within the city’s built-up area. This means that 68% of the total built-up area in Bytom (as at 2011) has been subject to land subsidence to a greater or lesser extent. It is for this reason, among others, that Bytom is one of the most rapidly depopulating cities in Poland and Europe, which in turn is a consequence of the closure of most industrial plants in the city after 1989^61,62^. An additional factor contributing to the decline in the city’s population has been the deteriorating technical condition of its buildings (especially old ones from the late 19th and early twentieth centuries). As a result of the damage to buildings, inhabitants of the Karb and Miechowice districts and of some areas in the city centre have been displaced. The districts affected by land subsidence have experienced the most rapid depopulation^63^.

**Figure 7:**
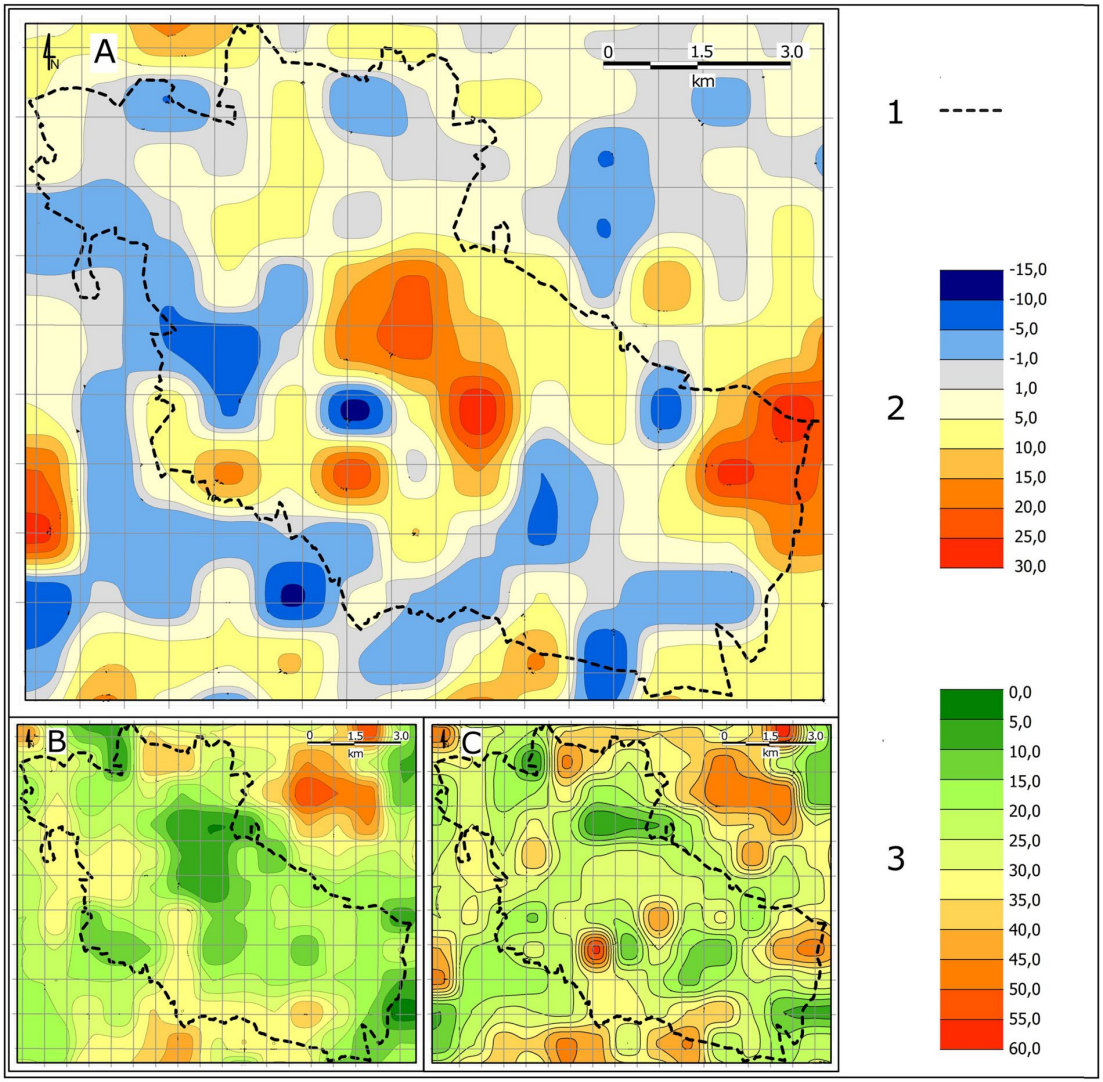
Changes in Bytom’s relief intensity in the nineteenth and twentieth centuries: A—changes in relative elevation from the late nineteenth century to the late twentieth century; B—variation in relative elevation in the late nineteenth century; C—variation in relative elevation in the late twentieth century; 1—city boundary; 2—magnitude of change in relative elevation from the late nineteenth century to the late twentieth century; 3—variation in relative elevation from the late nineteenth century to the late twentieth century.

The resulting subsidence basins within the city testify to the huge quantities of mineral resources that have been extracted (Fig. 7). Their extraction produced much greater quantities of waste (so-called waste rock), which was stored in heaps. Within the borders of Bytom, the increase in absolute elevation affected slightly more than 14% of the city area. Where the terrain became raised, its elevation rose most commonly by 1.1 m to 5.0 m. The largest heaps in terms of occupied area were located close to the eastern boundary of the city. Large waste rock dumps were also found in districts in the south-eastern part of the city. The highest absolute elevation of 35 m was reached by the heap situated in the south-western part of Bytom. Smaller heaps and dumps of waste rock and slag, which is a waste product generated in metal smelting processes, were also found in other districts of the city. A common phenomenon in recent years has been the backfilling of subsidence basins with waste rock, thus increasing land elevation in relation to sea level.

## Conclusions

As a result of human activity in the urban areas of Bytom, in the years 1883–2011 changes in minimum and maximum absolute elevations were found—from 250.0 to 243.0 m a.s.l. and from 340.0 to 348.4 m a.s.l., respectively. The difference between maximum and minimum elevation increased from 90.0 m to 105.4 m during that period.

A consequence of underground mining are extensive subsidence basins reaching 35 m in depth, which emerged within the city limits. In the period examined, the anthropogenic subsidence rate averaged 43 mm per year.

During the study period, differences in land elevations within the city became much more pronounced, which was reflected among others by a decrease in the share of land with gradients of up to 3° from 85.1 to 42.0% and an increase in the share of land with gradients above 9.1° from 0.8 to 15.8%. Changes in land gradients were caused by the appearance of the aforementioned subsidence basins, but also numerous heaps and dumps. Within the borders of Bytom, the increase in absolute elevation affected slightly more than 14% of the city area. Where the terrain became raised, its elevation rose most commonly by 1.1 m to 5.0 m, with maximum increase in elevation caused by human activity amounting to 35 m.

The presented method of assessing changes in elevation can be successfully applied to areas well documented on accurate and detailed topographic maps. The spectrum of its application may be considered in cognitive terms, e.g. the data obtained can be used in further local and regional studies on all components of the environment and also in order to create a global database on land relief changes in mining areas (which would in practice be representative of the Anthropocene, since it would reflect changes in relief in the period with the greatest human impact on the environment to date). The typical application of the method is related to its use by spatial planners in order to illustrate a possible impact of land relief changes on the destruction of the urban fabric.

## References

[1] Bąbel J, Braziewicz J, Jaskóła M, Kretschmer W, Pajek M, Semaniak J, Scharf A, Uhl T (2005). The radiocarbon dating of the neolithic flint mines at Krzemionki in central Poland. Nucl. Instrum. Methods Phys. Res. Sect. B Beam Interact. Mater. Atoms.

[2] Salazar D, Jackson D, Guendon JL, Salinas H, Morata D, Figueroa V, Manríquez G, Castro V (2011). Early evidence (ca. 12,000 BP) for iron oxide mining on the pacific coast of South America. Curr. Anthropol..

[3] Dodson J, Li X, Sun N, Atahan P, Zhou X, Liu H, Zhao K, Hu S, Yang Z (2014). Use of coal in the Bronze Age in China. Holocene.

[4] Towersa J, Bonda J, Evansb J, Mainlandc I, Montgomery J (2017). An isotopic investigation into the origins and husbandry of Mid-Late Bronze Age cattle from Grimes Graves, Norfolk. J. Archaeol. Sci. Rep..

[5] Clark G, Jacks D (2007). Coal and the industrial revolution, 1700–1869. Eur. Rev. Econ. Hist..

[6] Akcin H, Kutoglu HS, Kemaldere H, Deguchi T, Koksal E (2010). Monitoring subsidence effects in the urban area of Zonguldak Hardcoal Basin of Turkey by InSAR-GIS integration. Nat. Hazards Earth Syst. Sci..

[7] Can E, Kuscu S, Mekik C (2012). Determination of underground mining induced displacements using GPS observations in Zonguldak-Kozlu Hard Coal Basin. Int. J. Coal Geol..

[8] Wang J, Wang P, Qin Q, Wang H (2017). The effects of land subsidence and rehabilitation on soil hydraulic properties in a mining area in the Loess Plateau of China. CATENA.

[9] Fan T, Yan J, Wang S, Zhang B, Ruan S, Zhang M, Li S, Chen Y, Liu J (2012). Water quality variation of mining-subsidence lake during the initial stage: Cases study of Zhangji and Guqiao Mines. J. Coal Sci. Eng..

[10] Johnson KS (2004). Subsidence hazards due to evaporite dissolution in the United States. Environ. Geol..

[11] Lee DK, Mojtabai N, Lee HB, Song WK (2013). Assessment of the influencing factors on subsidence at abandoned coal mines in South Korea. Environ. Earth Sci..

[12] Mancini F, Stecchi F, Zanni M, Gabbianelli G (2009). Monitoring ground subsidence induced by salt mining in the city of Tuzla (Bosnia and Herzegovina). Environ. Geol..

[13] Marschalko M, Yilmaz I, Křístková V, Fuka M, Kubečka K, Bouchal T (2013). An indicative method for determination of the most hazardous changes in slopes of the subsidence basins in underground coal mining area in Ostrava (Czech Republic). Environ. Monit. Assess..

[14] Martinec P, Schejbalova B (2004). History and environmental impact of mining in the Ostravw-Karvina Coal Field (Upper Silesia Coal basin, Czech Republic). Geol. Belg..

[15] Singh KB (2007). Pot-hole subsidence in Son-Mahanadi Master Coal Basin. Eng. Geol..

[16] Dang VK, Nguyen TD, Dao NH, Duong TL, Dinh XV, Weber C (2021). Land subsidence induced by underground coal mining at Quang Ninh, Vietnam: Persistent scatterer interferometric synthetic aperture radar observation using Sentinel-1 data. Int. J. Remote Sens..

[17] Guzy A, Malinowska AA (2020). Assessment of the impact of the spatial extent of land subsidence and aquifer system drainage induced by underground mining. Sustainability.

[18] Sopata P, Stoch T, Wojcik A, Mrochen D (2020). Land surface subsidence due to mining-induced tremors in the upper Silesian coal basin (Poland)—case study. Remote Sens..

[19] Tiwari A, Narayan AB, Dwivedi R, Swadeshi A, Pasari S, Dikshit O (2020). Geodetic investigation of landslides and land subsidence: Case study of the Bhurkunda coal mines and the Sirobagarh landslide. Surv. Rev..

[20] Yuan YF, Li HZ, Zhang HJ, Zhang YW, Zhang XW (2020). Improving reliability of prediction results of mine surface subsidence of Northern Pei County for reusing land resources. Appl. Sci. Basel.

[21] Zheng LP, Zhu L, Wang W, Guo L, Chen BB (2020). Land subsidence related to coal mining in China revealed by L-band InSAR analysis. Int. J. Environ. Res. Public Health.

[22] Harnischmacher S (2010). Quantification of mining subsidence in the Ruhr District (Germany). Géomorphologie Relief, Processus, Environnement.

[23] Harnischmacher S, Zepp H (2014). Mining and its impact on the earth surface in the Ruhr District (Germany). Zeitschrift fur Geomorphologie.

[24] Machowski R, Rzetala MA, Rzetala M, Solarski M (2016). Geomorphological and hydro-logical effects of subsidence and land use change in industrial and urban areas. Land Degrad. Dev..

[25] Can E, Kuscu S, Kartal ME (2012). Effects of mining subsidence on masonry buildings in Zonguldak hard coal region in Turkey. Environ. Earth Sci..

[26] Szczepanska, J. & Twardowska, I. III.6—Mining waste. In *Solid Waste: Assessment, Monitoring and Remediation. Waste Management Series* (eds Twardowska, I. *et al.*), Vol. 4, 319–385, 10.1016/S0713-2743(04)80015-1 (2004).

[27] Nyssen J, Vermeersch D (2010). Slope aspect affects geomorphic dynamics of coal mining spoil heaps in Belgium. Geomorphology.

[28] Masoudian MS, Zevgolis IE, Deliveris AV, Marshal AM, Heron CM, Koukouzas NC (2019). Stability and characterisation of spoil heaps in European surface lignite mines: A state-of-the-art review in light of new data. Environ. Earth Sci..

[29] Abramowicz A, Rahmonov O, Chybiorz R (2020). Environmental management and landscape transformation on self-heating coal-waste dumps in the Upper Silesian Coal Basin. Land.

[30] Quanyuan W, Jiewu P, Shanzhong Q, Yiping L, Congcong H, Tingxiang L, Limei H (2009). Impacts of coal mining subsidence on the surface landscape in Longkou city, Shandong Province of China. Environ. Earth Sci..

[31] Abdikan S, Arikan M, Sanli FB, Cakirm Z (2014). Monitoring of coal mining subsidence in peri-urban area of Zonguldak city (NW Turkey) with persistent scatterer interferometry using ALOS-PALSAR. Environ. Earth Sci..

[32] Detailed Geological Map of Poland, 1:50,000 (Geological Publisher, 1954).

[33] Topographic map (Topographische karte), 1:25,000. Messtischblatt, 5679–Beuthen. Berlin (1934).

[34] Topographic map of Poland, 1: 10,000. (Chief Land Surveyor, 1994).

[35] Jankowski, W. German map at a scale of 1: 25,000 in the Polish territories east of the Odra and Nysa rivers. *Geodetic Rev.* **33**(12), 417–422 (**in Polish**) (1961).

[36] Konias A (2010). Topographic Cartography of the State and the Prussian Partition from the Second Half of the 18th Century to the Middle of the 20th Century.

[37] Skaloš J, Weber M, Lipský Z, Trpáková I, Šantrůčková M, Uhlířová L, Kukla P (2011). Using old military survey maps and orthophotograph maps to analyse long-term land cover changes—Case study (Czech Republic). Appl. Geogr..

[38] James A, Hodgson ME, Ghoshal S, Latiolais MM (2012). Geomorphic change detection using historic maps and DEM differencing: The temporal dimension of geospatial analysis. Geomorphology.

[39] Szypuła B (2020). Digital adaptation of the Geomorphological Map of Upper Silesian Industrial Region, Poland (1:50,000)—Old map new possibilities. J. Maps.

[40] Terrone M, Piana P, Paliaga G, D’Orazi M, Faccini F (2021). Coupling historical maps and LiDAR data to identify man-made landforms in urban areas. ISPRS Int. J. Geo-Inf..

[41] Henselowsky F, Rölkens J, Kelterbaum D, Bubenzer O (2021). Anthropogenic relief changes in a long-lasting lignite mining area (‘Ville’, Germany) derived from historic maps and digital elevation models. Earth Surf. Proc. Land..

[42] Deng J, Harff J, Giza A, Hartleib J, Dudzińska-Nowak J, Bobertz B, Furmańczyk K, Harff J, Furmańczyk K, von Storch H (2017). Reconstruction of coastline changes by the comparisons of historical maps at the Pomeranian Bay, Southern Baltic Sea. Coastline Changes of the Baltic Sea from South to East Coastal Research Library.

[43] Dulias, R. The Impact of Mining on the Landscape, A Study of the Upper Silesian Coal Basin in Poland. Environmental Science and Engineering. 209, (Springer International Publishing Switzerland, 2016).

[44] Habib M (2021). Evaluation of DEM interpolation techniques for characterizing terrain roughness. CATENA.

[45] Wawrzynkiewicz W, Sablik J (2002). Organic sulphur in the hard coal of the stratigraphic layers of the Upper Silesian coal basin. Fuel.

[46] Szypuła B (2014). Quantitative changes of anthropogenic relief over the last 100 years in the Silesian Upland (south Poland). Z. Geomorphol..

[47] Cabala JM, Cmiel SR, Idziak AF (2004). Environmental impact of mining activity in the Upper Silesian Coal Basin (Poland). Geol. Belg..

[48] Dwucet K, Wach J, Dominik A (1994). Calculation of land surface changes caused by deep mining exploitation on the example of Katowice voivodeship. A guide to Exercises in Environmental Protection.

[49] Perski Z, Jura D (1999). ERS SAR interferometry for the land subsidence detection in coal mining areas. Earth Observ. Q..

[50] Perski, Z. The use of satellite radar interferometry to determine the dynamics and extent of mining terrain deformations on the example of selected areas of the Upper Silesian Coal Basin, 9–39, (Faculty of Earth Sciences–University of Silesia, 2000). (**in Polish**).

[51] Machowski R, Rzetala MA (2014). Morpho- and hydrogenesis of water bodies in subsidence basins as exemplified by water bodies in Zabrze, Upper Silesia (Southern Poland). Z. Geomorphol..

[52] Rzetala, M. A. Morphogenesis of reservoirs in a mining-subsidence zone (a case study of the Bobrek river basin, southern Poland). In *13th International Multidiscyplinary Scientific Geoconference SGEM 2013*, Bulgaria, Vol. III, 237–244. 10.5593/SGEM2013/BC3/S12.030 (2013).

[53] Solarski M (2013). Anthropogenic transformations of the Bytom area relief in the period of 1883–1994. Environ. Socio-econ. Stud..

[54] Easterbrook DJ (1993). Surface Processes and Landforms.

[55] Adler FA, Tanner CCJ (2013). Urban Ecosystems. Ecological Principles for the Built Environment.

[56] Wang HM, Wang Y, Jiao X, Qian GR (2014). Risk management of land subsidence in Shanghai. Desalin. Water Treat..

[57] Dai, L. Preventing and Controlling Land Subsidence in Shanghai—towards more integrated and effective land use and ground water governance in the Yangtze Delta. http://meetingorganizer.copernicus.org/EGU2016/EGU2016-16451.pdf (Accessed 5 May 2019) (2016).

[58] Chaussard E, Amelung F, Abidin H, Hong SH (2013). Sinking Cities in Indonesia: ALOS-PALSAR detects rapid subsidence due to groundwater and gas extraction. Remote Sens. Environ..

[59] Kaneko S, Toyota T, Taniguchi M (2011). Long-term urbanization and land subsidence in Asian megacities: an indicators system approach. Groundwater and Subsurface Environments: Human Impacts in Asian Coastal Cities.

[60] Przyłucka M, Herrera G, Graniczny M, Colombo D, Béjar-Pizarro M (2015). Combination of conventional and advanced DInSAR to monitor very fast mining subsidence with TerraSAR-X data: Bytom City (Poland). Remote Sens..

[61] Bernt M, Haase A, Großmann K, Cocks M, Couch C, Cortese C, Krzysztofik R (2014). How does(n’t) urban shrinkage get onto the agenda? Experiences from Leipzig, Liverpool, Genoa and Bytom. Int. J. Urban Reg. Res..

[62] Rink D, Couch C, Haase A, Krzysztofik R, Rumpel P (2014). The governance of urban shrinkage in cities of post-socialist Europe: Policies, strategies and actors. Urban Res. Pract..

[63] Kantor-Pietraga I (2021). Does one decade of urban policy for the shrinking city make visible progress in urban re-urbanization? A case study of Bytom, Poland. Sustainability.

